# Shared Genetic Susceptibility Between Asthma and Immune‐Mediated Inflammatory Diseases

**DOI:** 10.1155/carj/4534431

**Published:** 2026-05-30

**Authors:** Liping Zhou, Kai Sun

**Affiliations:** ^1^ Department of Respiratory and Critical Care Medicine, Zigong Fourth People’s Hospital, Zigong, China, zg120.cn

**Keywords:** asthma, genetic correlations, genome-wide association study, immune-mediated inflammatory diseases (IMIDs)

## Abstract

**Background:**

Existing studies lack comprehensive evaluations of the genetic relationships between asthma and a broader spectrum of immune‐mediated inflammatory diseases (IMIDs). This study aims to elucidate the genetic correlation and potential causal interplay between asthma and IMIDs, thereby advancing insights into their shared genetic underpinnings.

**Methods:**

Using genome‐wide association study (GWAS) data, we investigated the shared genetic relationships between asthma and IMIDs through linkage disequilibrium score regression, multitrait GWAS analysis, and genotype‐tissue expression analysis.

**Results:**

Among 19 IMIDs, we identified significant genetic correlations between asthma and 13 IMIDs, with the strongest correlation observed with allergic rhinitis (rg = 0.665, *p* = 8.79 × 10^−27^). Multitrait GWAS analysis and Bayesian colocalization analysis identified 15 genetic loci with pleiotropic effects, of which nine loci exhibited shared causal relationships across multiple traits, which were predominantly clustered near genes associated with immune regulation and inflammatory response. Additionally, we demonstrated tissue‐ and cell‐type–specific enrichment of shared signals, with significant enrichment observed in the lung and spleen, as well as in B‐cells.

**Conclusions:**

Our findings reveal a significant genetic correlation between asthma and IMIDs. Further investigations are warranted to dissect the associations between these conditions and to elucidate the underlying mechanisms of the implicated loci.

## 1. Introduction

Asthma is a complex and heterogeneous disorder defined by chronic airway inflammation, heightened airway hyperresponsiveness, and reversible airflow obstruction, making it a major public health concern [1, 2]. The pathogenesis of asthma involves a complex interplay between genetic susceptibility and environmental factors [3]. Genome‐wide association studies (GWAS) have confirmed a significant genetic predisposition to asthma, with heritability estimated at 35%–90% [4, 5]. To date, more than 140 asthma susceptibility loci have been identified, many of which are associated with immune regulatory pathways (e.g., the IL‐13 signaling axis and the HLA region), inflammation, and airway remodeling [6–8].

In recent years, epidemiological studies have suggested that asthma often co‐occurs with atopic diseases, such as atopic dermatitis and allergic rhinitis, and may also exhibit comorbidities with immune‐mediated inflammatory diseases (IMIDs), such as rheumatoid arthritis and inflammatory bowel disease [9–12]. This comorbidity implies shared genetic susceptibility mechanisms across diseases, such as HLA‐DR3 gene, which has been found to be linked to a risk for autoimmune disease [13, 14]. Furthermore, genetic studies have revealed significant pleiotropy among IMIDs. For instance, studies have indicated shared risk loci between asthma and atopic dermatitis on chromosome 5 (TSLP region), potentially linked to dysregulation of Th2 immune responses [15, 16]. Additionally, polymorphisms in HLA Class II genes, which have been implicated in autoimmune disorders such as Type 1 diabetes and celiac disease, have also been found to correlate with asthma severity [17–19]. However, most existing studies focus on individual diseases or limited phenotypic associations, lacking comprehensive evaluations of the genetic relationships between asthma and a broader spectrum of IMIDs, with the functional pathways and therapeutic targets of shared genes remaining poorly understood.

Therefore, in this study, we leveraged large‐scale GWAS data (*N* = 7,765,172) to identify shared genetic factors between asthma and IMIDs, aiming to pinpoint shared single nucleotide variants (SNVs), genes, and pathways, providing valuable insights treating asthma patients with comorbid conditions.

## 2. Methods

### 2.1. Data Sources

In the present study, the GWAS summary statistics for asthma were derived from the FinnGen study, comprising 30,770 cases and 270,290 controls [20]. For IMIDs, including atopic dermatitis, allergic rhinitis, Type 1 diabetes, rheumatoid arthritis, alopecia areata, primary biliary cirrhosis, primary sclerosing cholangitis, vitiligo, ankylosing spondylitis, systemic lupus erythematosus, psoriasis, hypothyroidism, celiac disease, multiple sclerosis, inflammatory bowel disease, myasthenia gravis, irritable bowel syndrome, and Graves’ disease, the GWAS summary statistics were obtained from multiple sources: the UK Biobank, the FinnGen study, and the International Primary Sclerosing Cholangitis Study Group [20–22]. Comprehensive details of these GWAS datasets, including sample sizes and the primary source/definition for case identification, were documented in Supporting Table S1.

### 2.2. Linkage Disequilibrium Score Regression (LDSC)

To systematically evaluate the genetic correlation between asthma and IMIDs, we performed LDSC [23]. LDSC quantifies the polygenic‐driven genetic correlation (rg) between traits using summary statistics and accounts for potential sample overlap and cryptic relatedness by modeling the inflation in test statistics that is not explained by the polygenic effect. The genetic correlation (rg) ranges from −1 to 1, where −1 indicates a perfect inverse correlation and one indicates a perfect positive correlation. A false discovery rate (FDR)–adjusted *p* value < 0.05 was considered statistically significant.

### 2.3. Bidirectional Mendelian Randomization

To assess the potential bidirectional causal relationship between asthma and IMIDs, this study employed a bidirectional Mendelian randomization (MR) approach. This method utilized genetic variants as instrumental variables (IVs) to investigate the causal effect of asthma on IMIDs (forward analysis) and the causal effect of IMIDs on asthma (reverse analysis) through two independent analyses, thereby elucidating the temporal sequence and directionality of interactions between these diseases. The selection of instrumental variables must satisfy three core assumptions: Relevance assumption: SNPs must be strongly associated with the exposure (selection criteria: GWAS significance threshold *p* < 5 × 10^−8^ and F‐statistic > 10); Independence assumption: SNPs must be independent of confounding factors; Exclusion restriction: SNPs influence the outcome solely through the exposure, ruling out horizontal pleiotropy. Causal effect estimates were primarily derived using the inverse‐variance weighted method, with sensitivity analyses conducted using the weighted median, maximum likelihood, and MR‐Egger regression to ensure robustness of the results.

### 2.4. Cross‐Trait Meta‐Analysis

To systematically identify shared genetic loci and their functional mechanisms between asthma and IMIDs, we employed a multi‐stage genetic analysis strategy integrating cross‐trait meta‐analysis and functional annotation. Based on the asthma‐IMID disease pairs with significant genetic correlation identified in prior LD score regression analyses, we first conducted analysis of GWAS summary statistics using the MTAG (Multi‐trait analysis of GWAS) tool to identify of genetic loci shared across traits [24]. Using the genetic correlation matrix, MTAG assigned joint effect weights to each SNP and generated cross‐trait meta‐analysis *p*‐values (*P*_meta_). Genome‐wide significant loci were identified using a threshold of *P*_meta_ < 5 × 10^−8^. To validate loci with pleiotropic effects, we further applied CPASSOC (Cross‐phenotype association analysis) [25], which utilized a multi‐trait Z‐score matrix to construct the SHet statistic while employing LD score regression to control for sample overlap and population stratification. Only loci meeting both *P*_*m* *e* *t* *a*_ < 5 × 10^−8^ and single‐trait association *P*_single_ < 0.05 were retained. For the significant variants identified, functional annotation was performed using the ANNOVAR software [26].

### 2.5. Colocalization Analysis

We utilized the *R* package coloc to assess whether the association signals between asthma and IMIDs exhibit colocalization [27]. Based on the shared SNVs identified in the previous analysis, we extracted the variants within a 500 Kb around each SNV and calculated the posterior probability (PP.H4) that the two traits share a common causal variant. A posterior probability greater than 0.7 was considered evidence of colocalization. In addition, we employed the HyPrColoc package to estimate the posterior probability of multiple traits sharing the same causal SNV [28].

### 2.6. Gene‐Based Association Analysis

Subsequently, we applied four gene‐level analysis methods (Transcriptome‐wide association studies (TWAS)‐fusion [29], Summary‐data‐based Mendelian randomization (SMR) [30], multimarker analysis of genomic annotation (MAGMA) [31], and GCTA‐fastBAT [32]) to identify genes shared between asthma and IMIDs. In each method, *p* value thresholds were adjusted using FDR correction. TWAS integrate gene expression models with GWAS summary statistics to identify associations between gene expression levels and phenotypes. We performed TWAS analysis using the FUSION software based on precomputed expression models from the GTEx database (V8) across 49 tissue types [33]. SMR integrates GWAS data with eQTL data to examine pleiotropic associations between gene expression and diseases. This method uses genetic variants as instrumental variables, assuming that SNP effects on the phenotype are mediated through gene or molecular traits, while the HEIDI test is used to rule out LD confounding. For the SMR analysis, we used cis‐eQTL summary data from eQTLGen [34] and GTEx V8 data from four relevant tissues, including lung, skin (both sun‐exposed and not sun‐exposed), and whole blood. Genes associated with asthma‐trait pairs were defined as those with a *P*_*S* *M* *R*_ value passing the FDR correction threshold and a *P*_*H* *E* *I* *D* *I*_ > 0.01. MAGMA is a gene‐ and gene‐set–based association analysis method that integrates SNP effects using a multivariate regression framework to assess the overall association between genes and phenotypes. GCTA‐fastBAT is a variance‐component–based gene‐level analysis method that evaluates the overall association between genes and phenotypes by jointly analyzing the genetic effects of all SNPs within a gene. These four methods offer complementary insights: TWAS leverages gene expression data to infer associations, SMR tests for mediation by expression quantitative trait loci, MAGMA evaluates gene‐set enrichment, and GCTA‐fastBAT assesses variant effects aggregatively. Together, they capture diverse aspects of genetic architecture, reducing false negatives and enhancing robustness.

### 2.7. Tissue‐Specific Expression Analysis (TSEA) and Cell‐Type–Specific Enrichment Analysis

Based on the integration of the shared genes identified in the above analysis, a shared gene set was constructed. TSEA was performed on the shared genes to assess the enrichment of tissue‐specific genes within the input genes, which was conducted using deTS package, and *p* value thresholds were adjusted using FDR correction [35]. Cell‐type–specific enrichment analysis (CSEA) integrates single‐cell transcriptomic data with candidate gene lists to analyze the functional preferences of genes in specific cellular subpopulations. In this study, shared genes underwent CSEA using the WebCSEA tool [36]. A hypergeometric test was then performed to evaluate the significance of the enrichment of candidate genes within specific cell types.

## 3. Results

### 3.1. Genetic Correlation

**FIGURE 1:**
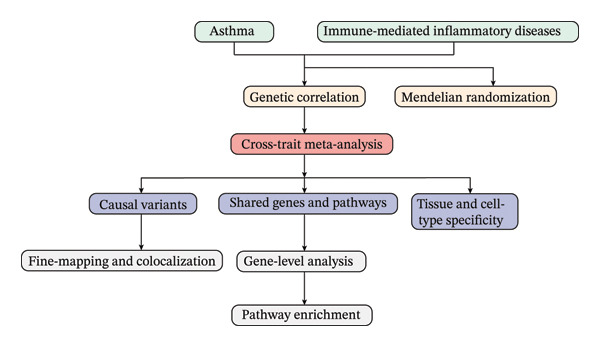
Schematic overview of the study design. The analysis involved acquiring GWAS summary statistics for asthma and immune‐mediated inflammatory diseases (IMIDs) from public databases, followed by genetic correlation, causal inference, and functional enrichment analyses to identify shared genetic architecture.

This study acquired GWAS summary statistics for 19 IMIDs (Figure 1). Utilizing these datasets, we elucidated the shared genetic architecture between asthma and IMIDs, including shared SNVs, genes, pathways, tissues, and cell types (Figure 1). LDSC analysis revealed positive genetic correlations between asthma and 13 IMIDs (Table 1). The strongest correlation was observed with allergic rhinitis (rg = 0.665, *p* = 8.79 × 10^−27^), followed by irritable bowel syndrome (rg = 0.522, *p* = 1.14 × 10^−21^). The weakest correlations emerged with celiac disease (rg = 0.126, *p* = 0.026) and inflammatory bowel disease (rg = 0.106, *p* = 0.016).

**TABLE 1:** Genetic correlations between asthma and different immune‐mediated inflammatory diseases.

trait1	trait2	rg	se	*p* value	FDR
Asthma	Allergic rhinitis	0.665	0.062	8.79e‐27	1.58e‐25
Asthma	Irritable bowel syndrome	0.522	0.055	1.14e‐21	1.02e‐20
Asthma	Atopic dermatitis	0.407	0.063	1.30e‐10	4.69e‐10
Asthma	Rheumatoid arthritis	0.349	0.046	2.50e‐14	1.50e‐13
Asthma	Alopecia areata	0.321	0.134	0.016	0.025
Asthma	Hypothyroidism	0.245	0.037	4.23e‐11	1.90e‐10
Asthma	Psoriasis	0.215	0.05	1.50e‐05	4.51e‐05
Asthma	Primary biliary cirrhosis	0.212	0.077	0.006	0.01
Asthma	Graves’ disease	0.199	0.058	5.73e‐04	0.001
Asthma	Multiple sclerosis	0.182	0.05	3.08e‐04	6.94e‐04
Asthma	Ankylosing spondylitis	0.145	0.04	2.77e‐04	6.94e‐04
Asthma	Vitiligo	0.128	0.131	0.328	0.347
Asthma	Celiac disease	0.126	0.057	0.026	0.035
Asthma	Myasthenia gravis	0.115	0.112	0.304	0.342
Asthma	Inflammatory bowel disease	0.106	0.044	0.016	0.025
Asthma	Type 1 diabetes	0.085	0.064	0.184	0.236
Asthma	Systemic lupus erythematosus	0.065	0.106	0.538	0.538
Asthma	Primary sclerosing cholangitis	−5.41e‐02	0.048	0.257	0.308

### 3.2. Bidirectional Mendelian Randomization

Given that many IMIDs share common risk factors, the observed genetic correlations may result from complex pleiotropic effects. To address potential confounding and establish causal inference, we implemented a bidirectional MR analysis. Our MR analysis provided evidence suggestive of bidirectional causal relationships 200 B between asthma and allergic rhinitis, atopic dermatitis, hypothyroidism, psoriasis, and rheumatoid arthritis (Figure S1; Table S2). Furthermore, the analysis suggested that asthma might be a causal risk factor for systemic lupus erythematosus. Conversely, genetic predisposition to Type 1 diabetes was indicative of a potential causal role in asthma development.

### 3.3. Cross‐Trait Loci and Causal Variants

Using MTAG and CPASSOC, we identified 478 SNVs that were commonly present across 13 pairs of traits. Among these, the largest number of shared SNVs was observed between asthma and rheumatoid arthritis (*N* = 66), followed by hypothyroidism (*N* = 62) (Figure S2). To further filter for shared causal SNVs, we first performed fine‐mapping using FM‐summary and subsequently conducted colocalization analysis across different traits using coloc [37]. As a result, we identified 40 causal variants shared by two traits. Additionally, we applied HyPrColoc, which led to the identification of 15 causal variants shared among multiple traits (Figure S3). Notably, only nine of these SNVs exhibited significant colocalization (Figure S4). These shared SNVs were predominantly clustered near genes associated with immune regulation and inflammatory responses (Table S3). For instance, IL4R and IL21R were the nearest genes to SNVs shared between asthma and five other traits, with rs76715626 identified as a causal variant. Similarly, rs2476601 in the PTPN22 gene was shared between asthma and five additional traits. Moreover, several shared causal variants were located near SH2B3 and BACH2, both of which are known to play crucial roles in immune regulation [38, 39].

**FIGURE 2:**
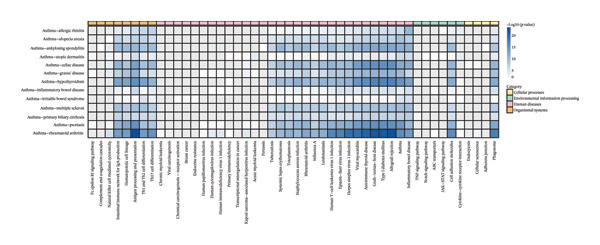
Enrichment of KEGG pathways among genes shared between asthma and IMIDs. Pathways are grouped by biological themes, with inclusion based on a hypergeometric test *p* < 0.05.

Subsequently, we conducted gene enrichment analysis for each pair of traits, revealing that their functional associations were primarily enriched in biological processes related to immune regulation, inflammatory responses, and intercellular communication (Figure 2). Immune‐related pathways, including Th1/Th2/Th17 cell differentiation, antigen presentation, and NK cell cytotoxicity, were found to play essential roles in maintaining the dynamic balance of autoimmune responses. Additionally, abnormalities in adhesion molecules and the intestinal IgA immune network were closely associated with mucosal barrier disruption and the imbalance between Th17 and Treg cells, further highlighting their potential contribution to disease pathogenesis.

### 3.4. Shared Genes and Pathways

**FIGURE 3:**
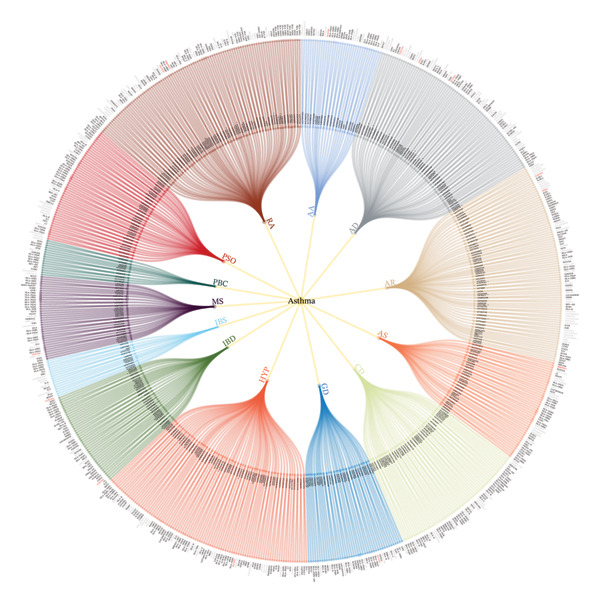
The circular dendrogram displays the shared loci between asthma and IMIDs. The inner ring shows the independent variants shared between asthma and IMIDs, with asterisks indicating shared causal variants with a posterior probability of H4 > 0.7. The outer ring displays the genes corresponding to the shared variants inferred through Annovar. Genes are highlighted in different colors to indicate their overlap with four gene identification methods (GCTA‐fastBAT, MAGMA, TWAS, and SMR): genes not identified by any method are shown in gray, those identified by at least one method are shown in black, and those identified by all four methods are shown in red.

Annotating GWAS variants solely based on gene proximity is an oversimplified approach that may fail to account for pleiotropy [40]. To address this limitation, we employed four complementary methods—TWAS‐Fusion, SMR, MAGMA, and GCTA‐fastBAT to infer shared genes (Figure S5). We defined disease‐associated genes as those identified by four methods, ultimately identifying a total of 127 genes (Table S4). By integrating the shared SNVs and genes, we constructed a comorbidity network for asthma, which systematically detailed the shared variants and genes for each pair of traits (Figure 3).

### 3.5. Tissue and Cell‐Type Specificity Analysis

Shared genes may play a functional role in certain specific tissues and cell types. TSEA and CSEA results showed significant enrichment in the lung and spleen (Figure S6), as well as in B‐cells, capillary endothelial cells, dendritic cells, endothelial cells, and innate lymphoid cells (Figure S7), across multiple asthma‐related traits, suggesting that these tissues and cell types may be key hubs in immune regulation. Additionally, we observed distinct shared patterns. For example, epithelial cells were only enriched in asthma and alopecia areata, while Langerhans cells were only enriched in asthma and rheumatoid arthritis.

## 4. Discussion

We discovered significant genetic correlations between asthma and IMIDs utilizing GWAS summary statistics in this study. Subsequent analyses uncovered shared pleiotropic variants, genes, biological pathways, and specific cell and tissue types associated with these traits, thus advancing our understanding of the common genetic underpinnings of asthma and IMIDs.

Extensive genetic associations were identified between asthma and IMIDs through the analysis of GWAS summary statistics. Among these, the association between asthma and allergic rhinitis was the most significant, with bidirectional causality observed between the two. Epidemiological studies have also reported numerous shared risk factors between asthma and allergic rhinitis, and these two diseases often co‐occur [41, 42]. Additionally, strong genome‐wide associations were found between asthma and alopecia areata, ankylosing spondylitis, irritable bowel syndrome, and inflammatory bowel disease. Although no causal relationships were detected, these findings suggest that other risk factors may have interfered with these associations.

This study identified several genes significantly associated with various immune‐related diseases, with their mechanisms primarily involving the regulation of Th2/Th17 immune responses, cytokine signaling pathways, and autoimmune susceptibility. IL4R and IL13 are core regulatory factors in Th2 immune responses and play a key role in asthma and atopic dermatitis [43]. IL4Rα, as a common receptor for IL‐4 and IL‐13, has genetic polymorphisms (e.g., rs2243250 and rs2070874) that are significantly associated with asthma risk [44]. IL‐13 induces airway mucus secretion and goblet cell hyperplasia through the activation of the STAT6 pathway, directly driving airway remodeling in asthma [45, 46]. Additionally, abnormal IL4R/IL‐13 signaling is closely linked to the pathogenesis of allergic rhinitis and eosinophilic esophagitis [47].

IL21R participates in the pathological processes of rheumatoid arthritis and psoriasis by regulating Th17 cell differentiation and B‐cell function [48, 49]. IL‐21 promotes the secretion of proinflammatory factors by synovial fibroblasts, exacerbating joint damage in RA, while also enhancing the cytotoxicity of CD8+ T‐cells, which is associated with inflammatory infiltration in psoriasis lesions. PTPN22 encodes lymphoid tyrosine phosphatase, and its loss‐of‐function mutations (e.g., rs2476601) significantly increase genetic susceptibility to rheumatoid arthritis, Type 1 diabetes, and Graves’ disease [50, 51]. This gene maintains immune tolerance by negatively regulating T‐cell receptor signaling, and its deficiency leads to excessive T‐cell activation and autoimmune attacks on self‐tissues [52]. As a transcription factor, BACH2 suppresses the transition of Th17 cells to a pathogenic phenotype by maintaining chromatin homeostasis [53]. BACH2‐deficient mice exhibit excessive Th17 cell activation, suggesting a protective role in multiple sclerosis and inflammatory bowel disease [54, 55].

SMAD3, a key molecule in the TGF‐β signaling pathway, exhibits promoter methylation, which is associated with an increased risk of asthma in offspring of mothers with asthma [56]. SH2B3 modulates lymphocyte development by negatively regulating the IL‐7R signaling pathway [57]. Variants of SH2B3 (e.g., rs3184504) are linked to genetic susceptibility to celiac disease and primary biliary cholangitis (PBC) [58, 59]. IRF1‐AS1, an antisense RNA of interferon regulatory factor 1, enhances IRF1 transcriptional activity, thereby modulating the Type I interferon pathway and playing a proinflammatory role in systemic lupus erythematosus and rheumatoid arthritis [60]. Polymorphisms in the IL7R gene (e.g., rs6897932) are strongly associated with the risk of multiple sclerosis, where they enhance Th17 cell survival and central nervous system infiltration, driving neuroinflammation [61, 62]. Collectively, the pleiotropic roles of these genes in immune regulation highlight shared molecular mechanisms underlying autoimmune and allergic diseases.

Additionally, our study has identified pathways associated with IMIDs. As a crucial component of innate immunity, the phagosome plays a key role in various immune‐related diseases. Its functional abnormalities may contribute to disease pathogenesis and progression by affecting pathogen clearance, inflammation regulation, and self‐antigen processing. In inflammatory bowel disease, upregulation of phagosome‐related pathways suggests that its dysfunction may contribute to disease onset by modulating immune responses and inflammatory processes [63]. In rheumatoid arthritis, the effects of TNF‐α inhibitors on macrophage phagosome maturation highlight the dual role of phagosome function in rheumatoid arthritis treatment. Activation of related signaling pathways may also be associated with inflammatory responses and disease progression in rheumatoid arthritis [64]. The pathogenesis of atopic dermatitis involves epidermal barrier defects and *Staphylococcus aureus* colonization. The role of the phagosome in pathogen clearance by skin macrophages and neutrophils may influence secondary infections and inflammatory responses in atopic dermatitis [65]. In autoimmune diseases, phagosome dysfunction may trigger autoimmunity through aberrant self‐antigen presentation, although direct evidence remains limited and requires further investigation.

It is important to note that while our MR analyses support potential causal relationships between asthma and several IMIDs, these inferences are contingent upon the validity of key assumptions inherent to the MR framework [66]. The instrumental variables (genetic variants) must be robustly associated with the exposure (relevance assumption), not associated with confounders (independence assumption), and influence the outcome solely through the exposure (exclusion restriction assumption). Although we employed sensitivity analyses to assess the robustness of our findings and mitigate bias from horizontal pleiotropy, the possibility of residual pleiotropy cannot be entirely ruled out. Furthermore, potential misclassification of complex phenotypes such as asthma in the original GWAS sources and shared upstream genetic mechanisms could also influence the results. Therefore, our findings should be interpreted as evidence supporting a causal hypothesis under specific genetic instrumental variable assumptions, rather than as definitive proof of causation. Future studies, including those with different designs and in diverse populations, are warranted to further validate these putative causal links.

Furthermore, as noted by previous studies leveraging publicly available GWAS summary data, heterogeneity in the definition of cases across different source datasets is an important consideration [67]. While the use of large‐scale consortium data provides substantial statistical power, differences in phenotypic specificity and accuracy could introduce heterogeneity. For instance, broader case definitions (e.g., self‐reported asthma) might capture a more genetically heterogeneous population compared to strict clinician‐diagnosed criteria, potentially influencing the magnitude of the observed genetic correlations. Although LDSC is generally robust to some forms of heterogeneity, such differences remain a potential source of bias that should be considered when interpreting our results. Future studies with more uniform and refined phenotyping across cohorts will be valuable for confirming and refining these findings.

This study has several limitations. First, the data were derived from public databases, and future research should conduct more comprehensive investigations into the underlying mechanisms of the identified loci. Second, the study population is primarily of European ancestry, which may limit the generalizability of the findings to other ethnic groups. Notably, asthma and IMIDs exhibit substantial differences in incidence, genetic architecture, and environmental exposures across ethnicities [68]. Thus, our results may not translate directly to non‐European cohorts. Future replication in diverse populations—including African, Asian, and Hispanic cohorts—is essential to validate the shared genetic mechanisms identified here and ensure broader applicability. Additionally, this study was unable to assess the impact of genetic variations on asthma subtypes. Despite these limitations, this study represents the largest‐scale analysis to date of the shared genetic relationships between asthma and various immune‐mediated inflammatory diseases.

## 5. Conclusions

In conclusion, this study demonstrates a strong genetic correlation between asthma and IMIDs and identifies a series of SNPs significantly associated with both traits. These findings highlight the shared genetic architecture between asthma and IMIDs, providing insights into shared biological mechanisms, which may guide future research on comorbid disease pathogenesis.

**Nomenclature**
IMIDs: Immune‐mediated inflammatory diseases
GWAS: Genome‐wide association study
SNVs: Single nucleotide variants
LD: Linkage disequilibrium
SNPs: Single nucleotide polymorphisms
MR: Mendelian randomization
IVs: Instrumental variables
MTAG: Multitrait analysis of GWAS
CPASSOC: Cross‐phenotype association analysis
TWAS: Transcriptome‐wide association studies
SMR: Summary‐data–based Mendelian randomization
MAGMA: Multimarker analysis of genomic annotation
TSEA: Tissue‐specific expression analysis
CSEA: Cell‐type–specific enrichment analysis

## Author Contributions

Liping Zhou and Kai Sun conceived the study and drafted the manuscript. Liping Zhou curated data and analyzed the data and participated in the data preparation and provided important comments on the manuscript. Kai Sun revised and approved the final version of the manuscript. The corresponding author attests that all listed authors meet authorship criteria and that no others meeting the criteria have been omitted.

## Funding

This work was supported by the Zigong Key Science and Technology Program (Grant No. 2020MZGC10).

## Disclosure

All authors have completed the ICMJE uniform disclosure form at http://www.icmje.org/disclosure-of-interest/and declare no support from any organization for the submitted work; no financial relationships with any organizations that might have an interest in the submitted work in the previous 3 years; no other relationships or activities that could appear to have influenced the submitted work.

## Ethics Statement

This study does not contain personal or medical information about an identifiable living individual, and animal subjects were not involved in the study.

## Consent

The authors have nothing to report.

## Conflicts of Interest

The authors declare no conflicts of interest.

## Supporting Information

Additional supporting information can be found online in the Supporting Information section.

## Supporting information


**Supporting Information** Figure S1: Causal inference between asthma and IMIDs. Causal inference was performed using two‐sample Mendelian randomization analysis with five methods (only statistically significant results are shown). In the figure, dots represent the odds ratios (ORs), color bars indicate the ±95% confidence intervals, and *p* values are displayed above the bars. Figure S2: Number of shared SNVs between asthma and IMIDs via MTAG and CPASSOC. Figure S3: Causal variants are shared by multiple traits, as identified by HyPrColoc. Figure S4: Colocalization plot of nine causal variants associated with asthma and IMIDs. Figure S5: Number of genes for each trait pair identified by four methods: GCTA, MAGMA, TWAS, and SMR. Each method is represented by one color. The numbers of identified genes are marked on each tier. Figure S6: Tissue‐specific expression analysis results. Figure S7: Cell‐type–specific enrichment analysis results. Blue bars represent significant enrichment (*p* value < 0.05). Table S1: Summary of GWAS data. Table S2: Causal inference between asthma and different immune‐mediated inflammatory diseases by two‐sample Mendelian randomization. Table S3: Cross‐trait meta‐analysis between asthma and immune‐mediated inflammatory diseases. Table S4: The list of asthma‐trait pair‐related genes identified by four gene‐based analyses.

## Data Availability

This paper analyzes existing, publicly available data. These access URLs for the datasets are listed in Table S1. This paper does not report the original code. Any additional information required to reanalyze the data reported in this paper is available from the lead contact upon request.
