# Supplementary figures and images for: Shared Genetic Susceptibility Between Asthma and Immune‐Mediated Inflammatory Diseases
Source: Can Respir J. 2026 May 30;2026:4534431. doi: 10.1155/carj/4534431 (PMC13239057; doi:10.1155/carj/4534431)

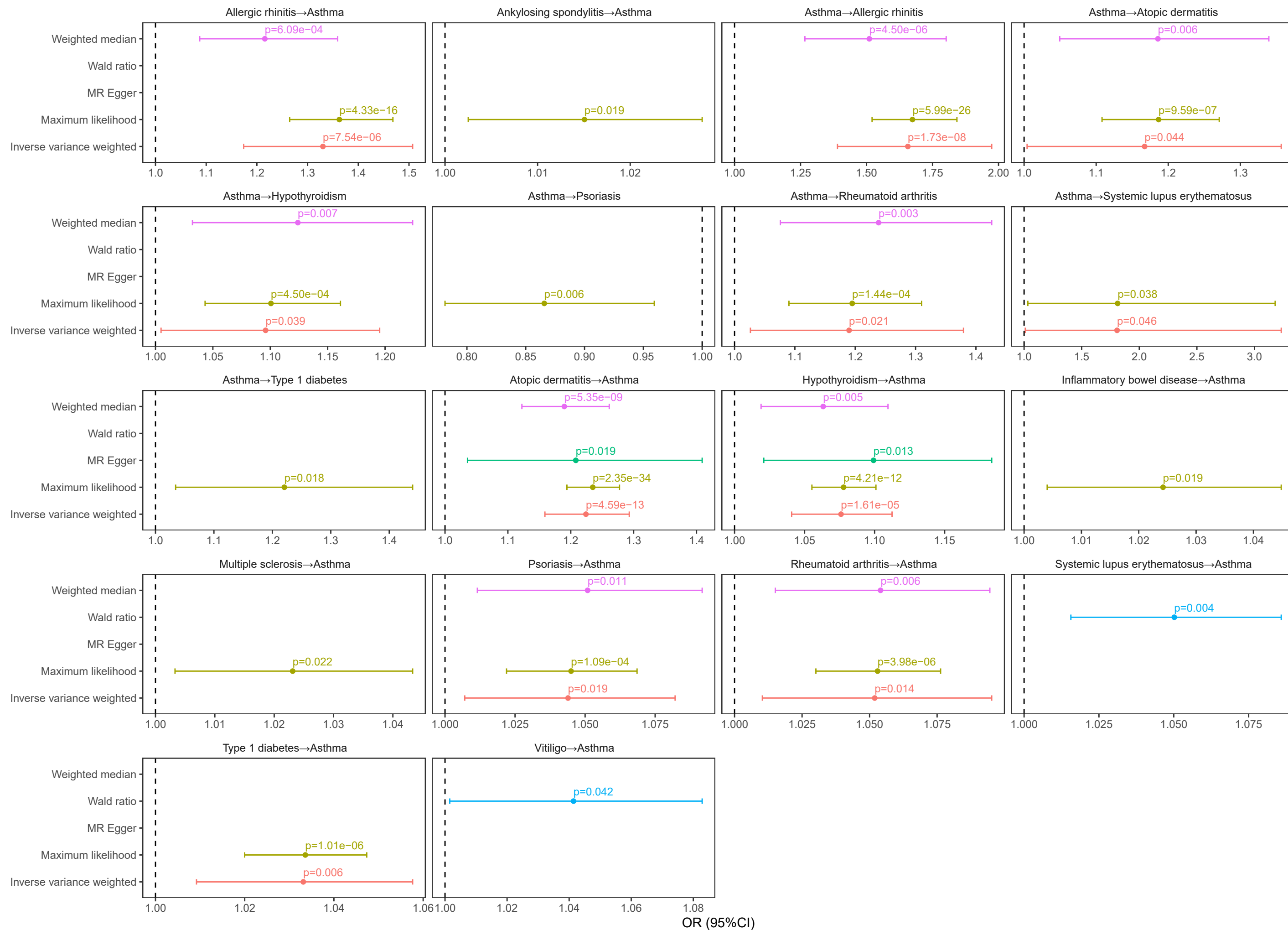

Supplement: Supplementary file 1 — Supporting Information Figure S1: Causal inference between asthma and IMIDs. Causal inference was performed using two‐sample Mendelian randomization analysis with five methods (only statistically significant results are shown). In the figure, dots represent the odds ratios (ORs), color bars indicate the ±95% confidence intervals, and p values are displayed above the bars. Figure S2: Number of shared SNVs between asthma and IMIDs via MTAG and CPASSOC. Figure S3: Causal variants are shared by multiple traits, as identified by HyPrColoc. Figure S4: Colocalization plot of nine causal variants associated with asthma and IMIDs. Figure S5: Number of genes for each trait pair identified by four methods: GCTA, MAGMA, TWAS, and SMR. Each method is represented by one color. The numbers of identified genes are marked on each tier. Figure S6: Tissue‐specific expression analysis results. Figure S7: Cell‐type–specific enrichment analysis results. Blue bars represent significant enrichment (p value < 0.05). Table S1: Summary of GWAS data. Table S2: Causal inference between asthma and different immune‐mediated inflammatory diseases by two‐sample Mendelian randomization. Table S3: Cross‐trait meta‐analysis between asthma and immune‐mediated inflammatory diseases. Table S4: The list of asthma‐trait pair‐related genes identified by four gene‐based analyses. [file CARJ-2026-4534431-s001.zip › Figure S1.pdf]

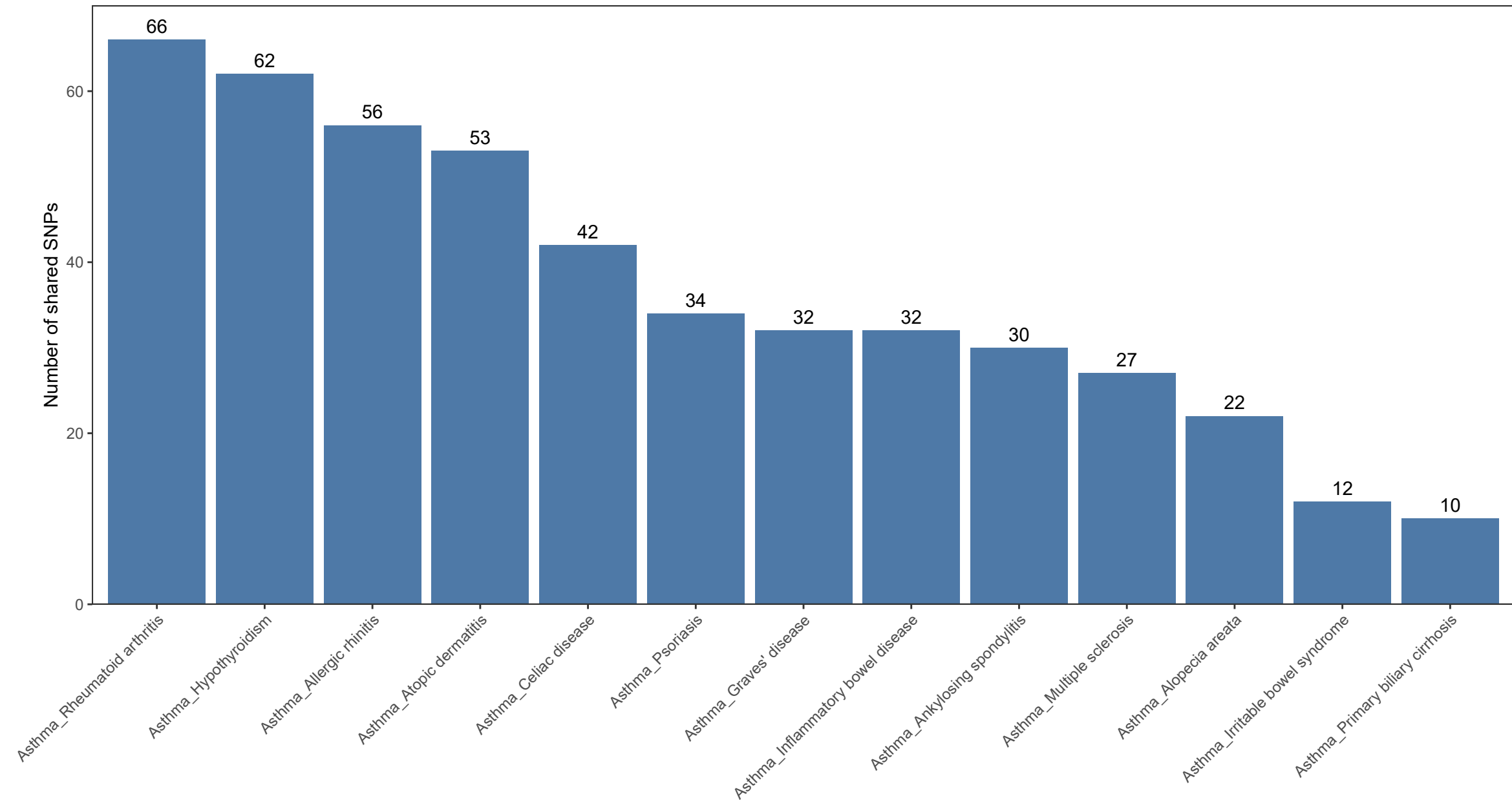

Supplement: Supplementary file 1 — Supporting Information Figure S1: Causal inference between asthma and IMIDs. Causal inference was performed using two‐sample Mendelian randomization analysis with five methods (only statistically significant results are shown). In the figure, dots represent the odds ratios (ORs), color bars indicate the ±95% confidence intervals, and p values are displayed above the bars. Figure S2: Number of shared SNVs between asthma and IMIDs via MTAG and CPASSOC. Figure S3: Causal variants are shared by multiple traits, as identified by HyPrColoc. Figure S4: Colocalization plot of nine causal variants associated with asthma and IMIDs. Figure S5: Number of genes for each trait pair identified by four methods: GCTA, MAGMA, TWAS, and SMR. Each method is represented by one color. The numbers of identified genes are marked on each tier. Figure S6: Tissue‐specific expression analysis results. Figure S7: Cell‐type–specific enrichment analysis results. Blue bars represent significant enrichment (p value < 0.05). Table S1: Summary of GWAS data. Table S2: Causal inference between asthma and different immune‐mediated inflammatory diseases by two‐sample Mendelian randomization. Table S3: Cross‐trait meta‐analysis between asthma and immune‐mediated inflammatory diseases. Table S4: The list of asthma‐trait pair‐related genes identified by four gene‐based analyses. [file CARJ-2026-4534431-s001.zip › Figure S2.pdf]

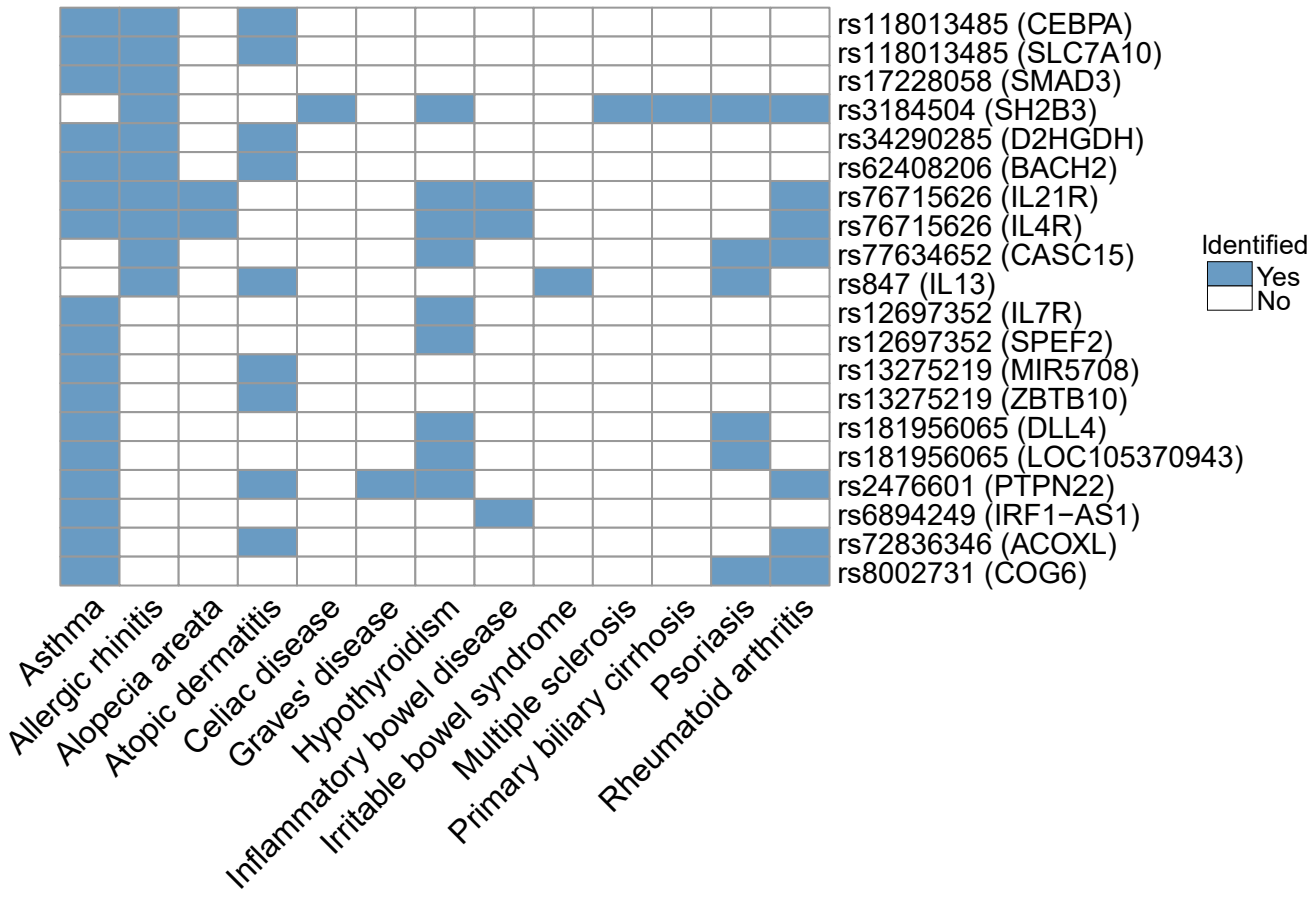

Supplement: Supplementary file 1 — Supporting Information Figure S1: Causal inference between asthma and IMIDs. Causal inference was performed using two‐sample Mendelian randomization analysis with five methods (only statistically significant results are shown). In the figure, dots represent the odds ratios (ORs), color bars indicate the ±95% confidence intervals, and p values are displayed above the bars. Figure S2: Number of shared SNVs between asthma and IMIDs via MTAG and CPASSOC. Figure S3: Causal variants are shared by multiple traits, as identified by HyPrColoc. Figure S4: Colocalization plot of nine causal variants associated with asthma and IMIDs. Figure S5: Number of genes for each trait pair identified by four methods: GCTA, MAGMA, TWAS, and SMR. Each method is represented by one color. The numbers of identified genes are marked on each tier. Figure S6: Tissue‐specific expression analysis results. Figure S7: Cell‐type–specific enrichment analysis results. Blue bars represent significant enrichment (p value < 0.05). Table S1: Summary of GWAS data. Table S2: Causal inference between asthma and different immune‐mediated inflammatory diseases by two‐sample Mendelian randomization. Table S3: Cross‐trait meta‐analysis between asthma and immune‐mediated inflammatory diseases. Table S4: The list of asthma‐trait pair‐related genes identified by four gene‐based analyses. [file CARJ-2026-4534431-s001.zip › Figure S3.pdf]

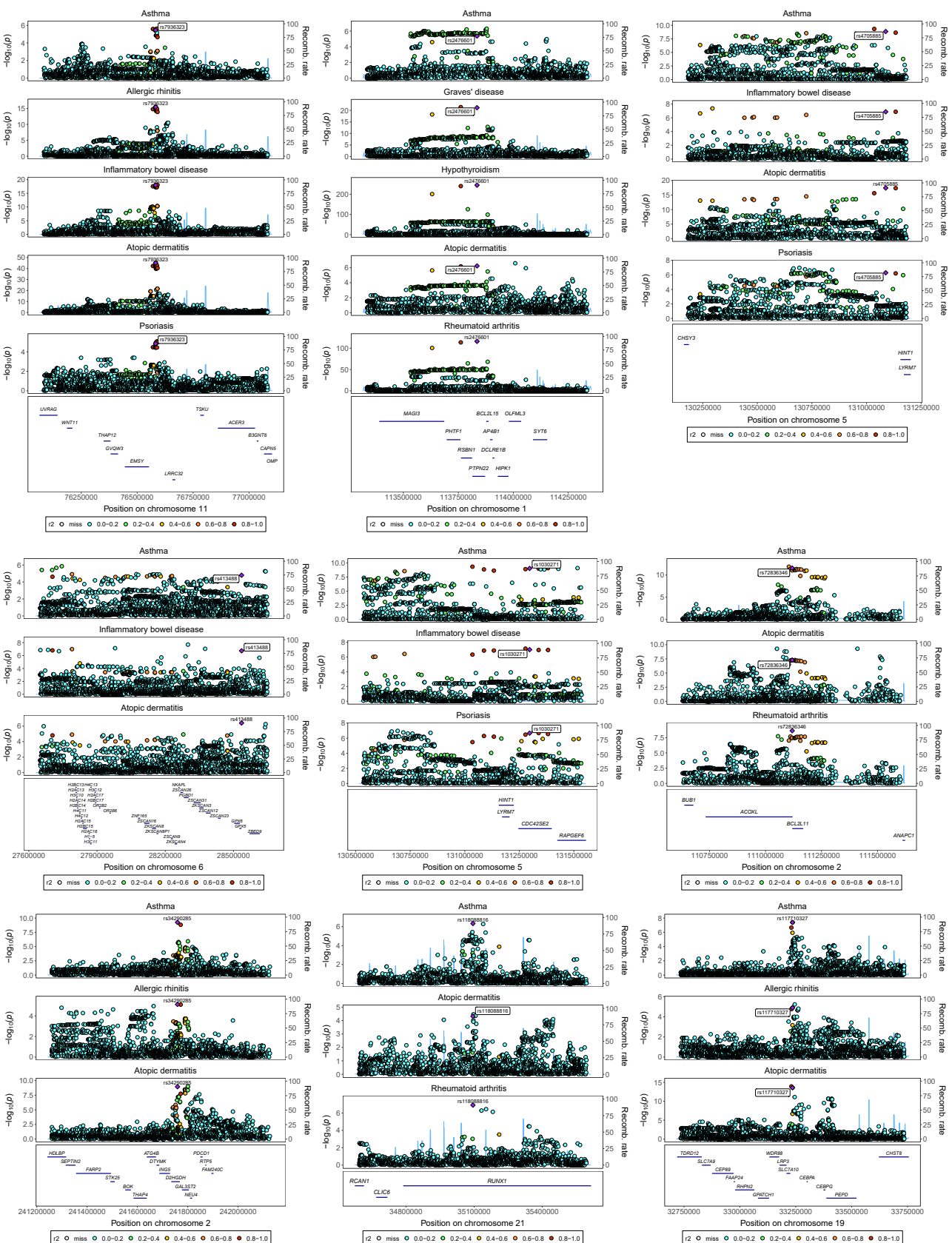

Supplement: Supplementary file 1 — Supporting Information Figure S1: Causal inference between asthma and IMIDs. Causal inference was performed using two‐sample Mendelian randomization analysis with five methods (only statistically significant results are shown). In the figure, dots represent the odds ratios (ORs), color bars indicate the ±95% confidence intervals, and p values are displayed above the bars. Figure S2: Number of shared SNVs between asthma and IMIDs via MTAG and CPASSOC. Figure S3: Causal variants are shared by multiple traits, as identified by HyPrColoc. Figure S4: Colocalization plot of nine causal variants associated with asthma and IMIDs. Figure S5: Number of genes for each trait pair identified by four methods: GCTA, MAGMA, TWAS, and SMR. Each method is represented by one color. The numbers of identified genes are marked on each tier. Figure S6: Tissue‐specific expression analysis results. Figure S7: Cell‐type–specific enrichment analysis results. Blue bars represent significant enrichment (p value < 0.05). Table S1: Summary of GWAS data. Table S2: Causal inference between asthma and different immune‐mediated inflammatory diseases by two‐sample Mendelian randomization. Table S3: Cross‐trait meta‐analysis between asthma and immune‐mediated inflammatory diseases. Table S4: The list of asthma‐trait pair‐related genes identified by four gene‐based analyses. [file CARJ-2026-4534431-s001.zip › Figure S4.pdf]

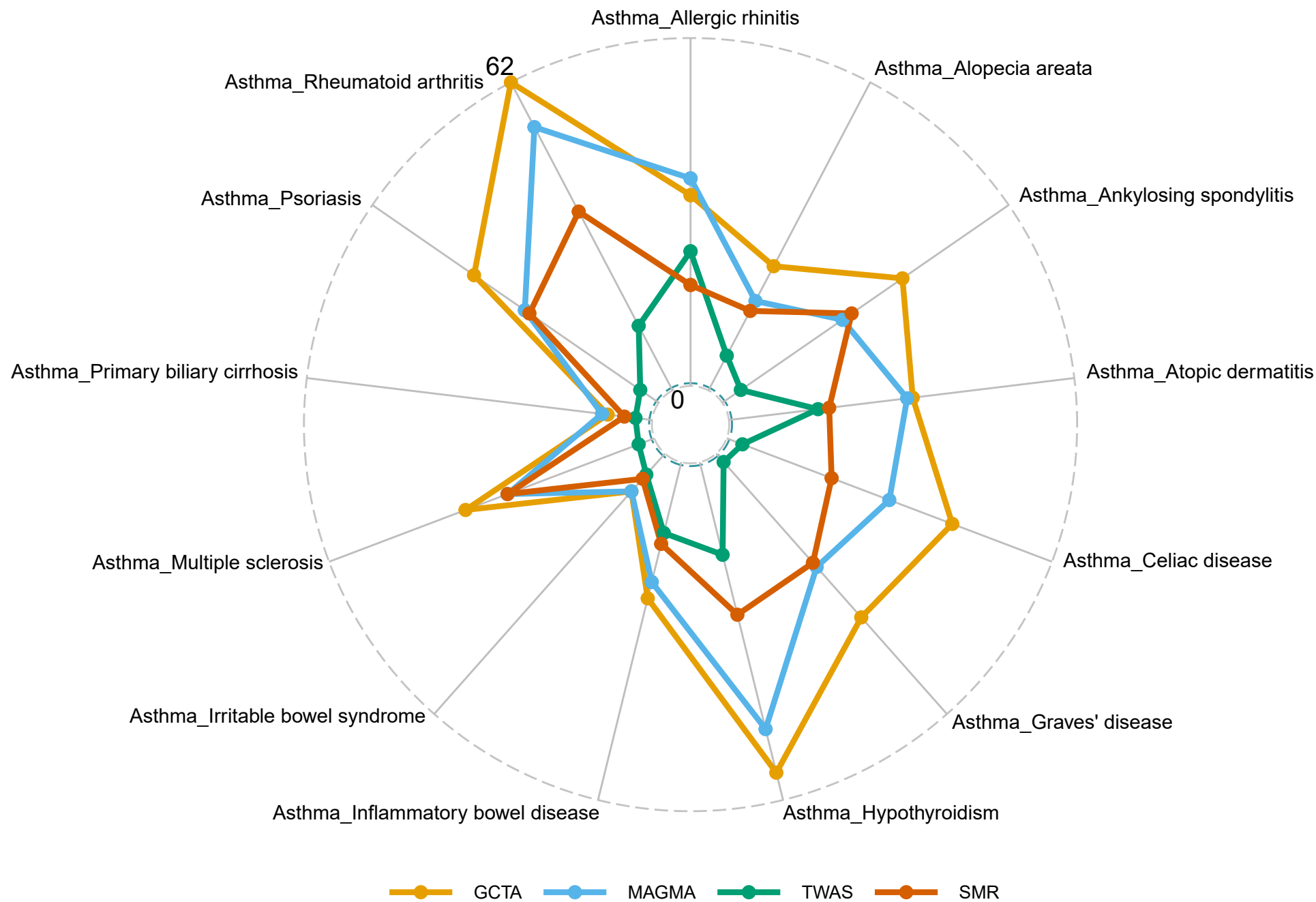

Supplement: Supplementary file 1 — Supporting Information Figure S1: Causal inference between asthma and IMIDs. Causal inference was performed using two‐sample Mendelian randomization analysis with five methods (only statistically significant results are shown). In the figure, dots represent the odds ratios (ORs), color bars indicate the ±95% confidence intervals, and p values are displayed above the bars. Figure S2: Number of shared SNVs between asthma and IMIDs via MTAG and CPASSOC. Figure S3: Causal variants are shared by multiple traits, as identified by HyPrColoc. Figure S4: Colocalization plot of nine causal variants associated with asthma and IMIDs. Figure S5: Number of genes for each trait pair identified by four methods: GCTA, MAGMA, TWAS, and SMR. Each method is represented by one color. The numbers of identified genes are marked on each tier. Figure S6: Tissue‐specific expression analysis results. Figure S7: Cell‐type–specific enrichment analysis results. Blue bars represent significant enrichment (p value < 0.05). Table S1: Summary of GWAS data. Table S2: Causal inference between asthma and different immune‐mediated inflammatory diseases by two‐sample Mendelian randomization. Table S3: Cross‐trait meta‐analysis between asthma and immune‐mediated inflammatory diseases. Table S4: The list of asthma‐trait pair‐related genes identified by four gene‐based analyses. [file CARJ-2026-4534431-s001.zip › Figure S5.pdf]

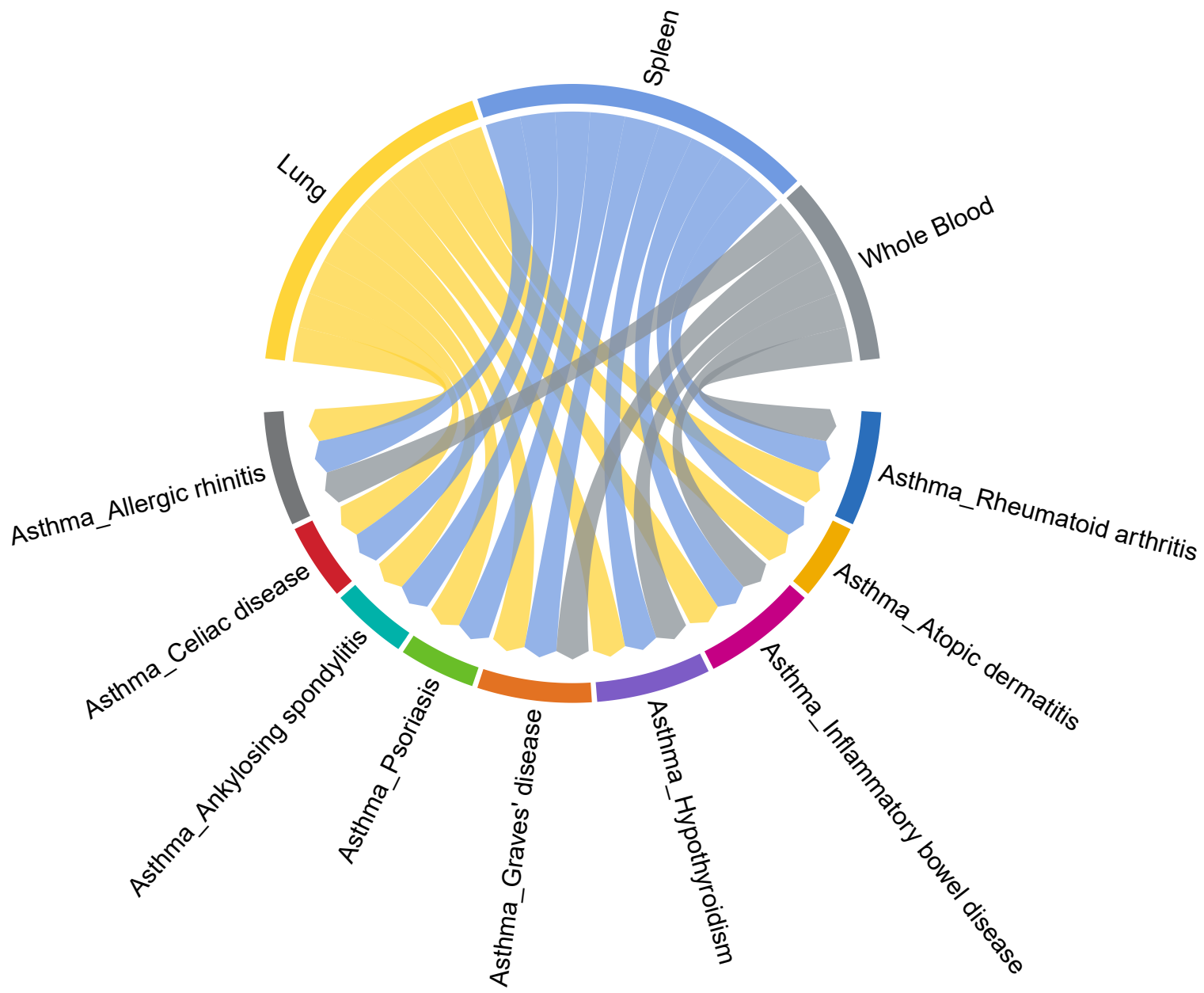

Supplement: Supplementary file 1 — Supporting Information Figure S1: Causal inference between asthma and IMIDs. Causal inference was performed using two‐sample Mendelian randomization analysis with five methods (only statistically significant results are shown). In the figure, dots represent the odds ratios (ORs), color bars indicate the ±95% confidence intervals, and p values are displayed above the bars. Figure S2: Number of shared SNVs between asthma and IMIDs via MTAG and CPASSOC. Figure S3: Causal variants are shared by multiple traits, as identified by HyPrColoc. Figure S4: Colocalization plot of nine causal variants associated with asthma and IMIDs. Figure S5: Number of genes for each trait pair identified by four methods: GCTA, MAGMA, TWAS, and SMR. Each method is represented by one color. The numbers of identified genes are marked on each tier. Figure S6: Tissue‐specific expression analysis results. Figure S7: Cell‐type–specific enrichment analysis results. Blue bars represent significant enrichment (p value < 0.05). Table S1: Summary of GWAS data. Table S2: Causal inference between asthma and different immune‐mediated inflammatory diseases by two‐sample Mendelian randomization. Table S3: Cross‐trait meta‐analysis between asthma and immune‐mediated inflammatory diseases. Table S4: The list of asthma‐trait pair‐related genes identified by four gene‐based analyses. [file CARJ-2026-4534431-s001.zip › Figure S6.pdf]

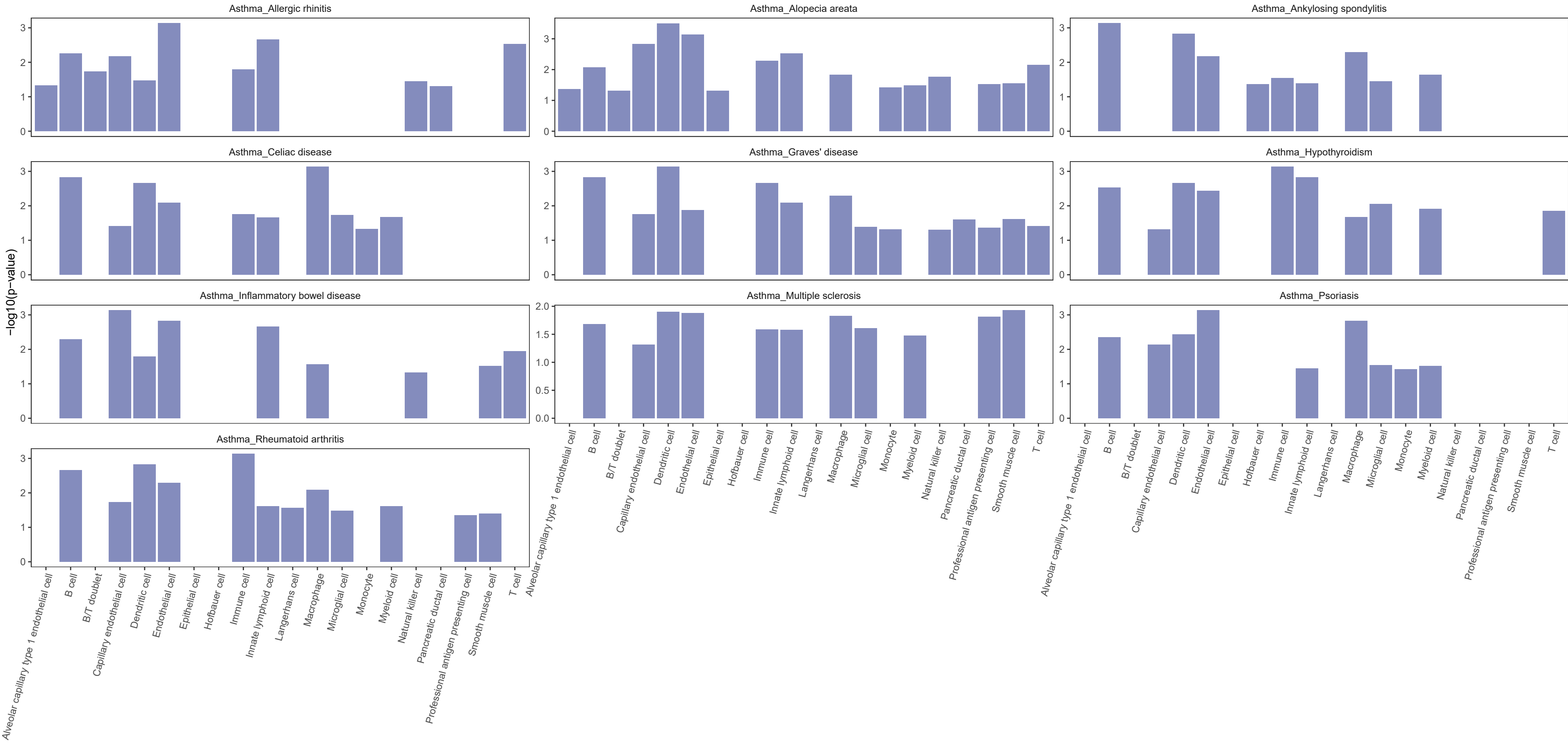

Supplement: Supplementary file 1 — Supporting Information Figure S1: Causal inference between asthma and IMIDs. Causal inference was performed using two‐sample Mendelian randomization analysis with five methods (only statistically significant results are shown). In the figure, dots represent the odds ratios (ORs), color bars indicate the ±95% confidence intervals, and p values are displayed above the bars. Figure S2: Number of shared SNVs between asthma and IMIDs via MTAG and CPASSOC. Figure S3: Causal variants are shared by multiple traits, as identified by HyPrColoc. Figure S4: Colocalization plot of nine causal variants associated with asthma and IMIDs. Figure S5: Number of genes for each trait pair identified by four methods: GCTA, MAGMA, TWAS, and SMR. Each method is represented by one color. The numbers of identified genes are marked on each tier. Figure S6: Tissue‐specific expression analysis results. Figure S7: Cell‐type–specific enrichment analysis results. Blue bars represent significant enrichment (p value < 0.05). Table S1: Summary of GWAS data. Table S2: Causal inference between asthma and different immune‐mediated inflammatory diseases by two‐sample Mendelian randomization. Table S3: Cross‐trait meta‐analysis between asthma and immune‐mediated inflammatory diseases. Table S4: The list of asthma‐trait pair‐related genes identified by four gene‐based analyses. [file CARJ-2026-4534431-s001.zip › Figure S7.pdf]
